# Seeing Is Believing: Using Video Feedback in Cognitive Therapy for Social Anxiety Disorder

**DOI:** 10.1016/j.cbpra.2016.03.007

**Published:** 2017-05

**Authors:** Emma Warnock-Parkes, Jennifer Wild, Richard Stott, Nick Grey, Anke Ehlers, David M. Clark

**Affiliations:** University of Oxford and King’s College London; University of Oxford; King’s College London; South London and Maudsley NHS Foundation Trust and King’s College London; University of Oxford

**Keywords:** video feedback, social anxiety disorder, cognitive therapy, processing biases

## Abstract

Distorted negative self-images and impressions appear to play a key role in maintaining Social Anxiety Disorder (SAD). In previous research, McManus et al. (2009) found that video feedback can help people undergoing cognitive therapy for SAD (CT-SAD) to develop a more realistic impression of how they appear to others, and this was associated with significant improvement in their social anxiety. In this paper we first present new data from 47 patients that confirms the value of video feedback. Ninety-eighty percent of the patients indicated that they came across more favorably than they had predicted after viewing a video of their social interactions. Significant reductions in social anxiety were observed during the following week and these reductions were larger than those observed after control periods. Comparison with our earlier data (McManus et al., 2009) suggests we may have improved the effectiveness of video feedback by refining and developing our procedures over time. The second part of the paper outlines our current strategies for maximizing the impact of video feedback. The strategies have evolved in order to help patients with SAD overcome a range of processing biases that could otherwise make it difficult for them to spot discrepancies between their negative self-imagery and the way they appear on video.

## Highlights

• Video feedback is an effective component of cognitive therapy for social anxiety disorder.
• Video feedback helps correct distorted negative self-images.
• Video feedback can also provide patients with insight into how their safety behaviors look to others.
• Social anxiety related processing biases can undermine the effects of video feedback if not addressed.
• Ways of circumventing the processing biases are described.

A recent network meta-analysis (Mayo-Wilson et al., 2014) has established that cognitive therapy for social anxiety disorder (CT-SAD) is an effective treatment that compares favorably with a range of other psychological and pharmacological interventions, including group CBT, exposure therapy, interpersonal psychotherapy, and psychodynamic psychotherapy. CT-SAD is based on the Clark and Wells (1995) cognitive model of SAD and involves a number of components: (1) developing a personalized cognitive model including the patient’s negative thoughts, self-images, focus of attention, safety behaviors and anxiety symptoms; (2) an experiential exercise to demonstrate the adverse effects of self-focused attention and safety behaviors; (3) video and still photograph feedback to correct negative self-imagery; (4) training in externally focused attention; (5) behavioral experiments to test patients’ negative beliefs by dropping safety behaviors and focusing attention externally in social situations and also by purposefully displaying feared behaviors or signs of anxiety (decatastrophizing); (5) surveys to discover other people’s view of feared outcomes; (6) memory work (discrimination training and memory rescripting) to reduce the impact of early social trauma experiences. Video feedback is a key component of the treatment and is used throughout therapy. This present-focused technique aims to counteract the distorted negative self-images that characterize social anxiety disorder (Hackmann, Surawy, & Clark, 1998) by helping patients to obtain a more realistic view of how they appear to other people. In this paper we will first describe a study providing updated evidence for the effectiveness of video feedback. Following this we will present clinical guidelines detailing a range of procedures for the successful implementation of video feedback.

McManus et al. (2009) reported on the effects of video feedback in the context of a standard course of CT-SAD. In 94% of patients, video feedback was associated with an improved appraisal of their performance. Significant reductions in social anxiety were observed in the week following video feedback and these exceeded those observed in a control week. A number of other studies have also demonstrated positive effects of video feedback in both clinical and subclinical samples (Harvey et al., 2000, Kim et al., 2002, Orr and Moscovitch, 2010, Parr and Cartwright-Hatton, 2009, Rapee and Hayman, 1996). However, two studies (Rodebaugh, 2004, Smits et al., 2006) failed to demonstrate beneficial effects of video feedback on social anxiety. In both studies, it appears that little time was devoted to preparing participants for viewing their videos and subsequent discussion of the viewing experience was also curtailed. These differences may help explain the negative findings. In an analogue study, Orr and Moscovitch (2010) found that detailed discussion following video viewing was essential in order to achieve substantial changes in both self-perception and subsequent social anxiety. The same authors (Orr & Moscovitch, 2013) also found video feedback to be less effective in socially anxious individuals who have additional concerns about their physical appearance. They propose that a preoccupation about physical appearance when viewing video may affect a patient’s ability to perceive that they come across better socially than they predicted they would. This is one example of the processing biases that can potentially undermine the effectiveness of video feedback. Over the years, we have noticed that patients with social anxiety disorder have a range of processing biases that make it difficult for them to see the difference between their habitual negative self-perception and the way they appear on video. To overcome these processing biases, we have developed detailed procedures for setting up video recordings, for preparing patients to view the recordings, and for discussing what they have seen.

The processing biases that can interfere with video feedback fall into five broad categories. First, *reexperiencing feelings when watching the video*. Clark and Wells (1995) hypothesized that patients with social anxiety disorder misleadingly use their feelings to decide how they appear in social interactions. When subsequently watching a video of a social interaction, many patients appear to reexperience some of their original feelings and these can become confused with the video image. As a consequence, they may rate themselves as coming across more negatively than other people who do not reexperience their feelings from the original interaction might have rated them. To prevent this bias from interfering, it is necessary to help patients to observe themselves as though they are observing a stranger, ignoring their feelings and only focusing on what would be visible to anyone.

Second, *selectively searching for behaviors that could be interpreted negatively.* Patients with SAD have a general belief that they come across badly in social interactions. This can lead them to selectively search a video for any behavior that could conceivably be interpreted in a negative fashion. This can happen even if they were not particularly concerned about those behaviors during the interaction and they were not prominent in the negative self-image. To solve this problem, it is necessary to ask patients to make clear predictions in advance of watching the video about any negative features that they believe were evident during the interaction.

Third, *discounting the accuracy of the video image.* If an anticipated negative feature (severe blushing, shaking, etc.) is not evident in the video, some patients may discount the accuracy of the video image, claiming that the camera is at fault (e.g., poor color rendition for blushing, or the shot was not zoomed in enough to pick up shaking). Therefore, it is necessary to carefully set up the recording so that patients are confident that features that they are concerned about will be visible if they occur. We call this procedure calibrating the video.

Fourth, *mistaking safety behaviors for social deficits.* Clark and Wells (1995) hypothesized that patients with SAD engage in a wide range of safety behaviors during social interactions in order to prevent their feared catastrophes from coming about. For example, individuals who are concerned that they may come across to others as uninteresting may say little. In addition, when they do speak, they may memorize what they have said and compare it with what they are about to say in order to check that it is sufficiently interesting. When watching the video, patients may see the observable side of these safety behaviors (appearing withdrawn and disengaged) and interpret this as an inherent social deficit. Asking patients whether they were intentionally holding back or doing other self-absorbing safety behaviors helps them realize that the apparent deficit in their social performance is the effect of a conscious strategy, which they can decide to drop in future.

Fifth, *reactivating habitual patterns of self-criticism.* During and after social interactions, patients with SAD are highly self-critical. When subsequently watching a video of the interaction, the self-critical commentary that accompanied or followed the interaction may be reactivated, making it difficult for patients to judge themselves objectively on the video. To address this, a number of techniques have been developed to reduce the reactivation of past memories and self-critical commentaries while viewing the video.

In the present paper, we first present data on the effectiveness of our current version of video feedback and compare it with the earlier version, whose effects were reported by McManus et al. (2009). Following this, we then describe in detail the clinical strategies that we now use to maximize the effects of video feedback. It is hoped that description of the strategies will help other clinicians to obtain optimal results when using video feedback.

## Effectiveness of Current Version of Video Feedback

Video feedback can be used in a variety of different ways during a course of CT-SAD. The first time that it is used is in Session 3. In the preceding session, patients engage in an experiential exercise in which they have a social interaction under two conditions: first, while focusing their attention on themselves, evaluating their performance, and engaging in habitual safety behaviors; second, while focusing externally, getting lost in the interaction (as opposed to evaluating it) and dropping their safety behaviors. Typically, they discover that they feel less anxious and think they come across better to other people in the second condition, a discovery that is built on in subsequent therapy. In Session 3, video feedback of both social interactions is used to help patients compare their predictions of how they think they came across with how they actually came across. McManus et al. (2009) found that video feedback in Session 3 helped 94% of patients to see that they came across to other people more positively than they had predicted.

Since the McManus study, improved availability and functionality of camera and smartphone technology has increased the options for using video feedback. Technology now makes it easy to capture as a still shot key moments that disconfirm patients’ negative beliefs, and we have found this to be a very useful technique for consolidating learning. Further experience in using video feedback has also made us more aware of some of the processing biases (outlined above) that can make it difficult for patients to fully benefit from viewing themselves on video and clinical strategies for overcoming these biases have been developed. In order to assess the impact of our current way of using video feedback, we present video feedback data from our most recent RCT (Clark et al., Trial registration: ISRCTN95458747) and compare it with that reported in the McManus et al. (2009) study.

## Method

### Participants

Participants were 47 patients who met *Diagnostic and Statistical Manual for Mental Disorders* (DSM-IV; American Psychiatric Association, 1994) criteria for SAD and were treated with individual cognitive therapy for SAD following the general protocol outlined in Clark et al. (2006). Other inclusion and exclusion criteria were identical to the Clark et al. (2006) trial, which provided the main data for the McManus et al. (2009) report. Participants’ mean age was 32 years (*SD* = 8.82). Forty-nine percent (23) were female.

### Procedure

Here we report the data from Session 3 of CT-SAD. In this session, participants viewed the video recordings of the two social interactions they had engaged in during Session 2. In most instances, the Session 2 interactions involved having a conversation with a stranger. However, if patients felt this would not elicit a significant degree of anxiety, an alternative individualized task was chosen, such as having a conversation with a small group of people. Prior to watching the videos of both interactions in Session 3, patients were asked to form a mental image of the way they thought they would appear and to make clear-cut predictions about how they would come across. The videos were then watched and discussed before patients rerated how they thought they appeared. Details of how predictions were generated with patients and how the video was viewed and discussed are given below in the clinical guidelines section.

### Measures

#### Self-Perception

Before watching each video, patients were asked to rate: (a) How anxious do you think you will look, on a scale ranging from 0 (*not anxious at all*) to 100 (*the most anxious you have ever felt*)?; (b) the extent to which idiosyncratic feared outcomes might be evident (e.g., How boring do you think you will look? [0–100]); and (c) How would you rate your performance overall (0–100)? These ratings were repeated after each video had been viewed and discussed.

#### Social Anxiety

Participants completed the self-report version of the Liebowitz Social Anxiety Scale (LSAS-SR; Baker, Heinrichs, Kim, & Hofmann, 2002) prior to each treatment session. The LSAS-SR has demonstrated good test–retest reliability and validity (Baker et al., 2002). Internal consistency in our sample was excellent (α = 0.92).

### Results

#### Effect of Video Feedback on Self-Perception

Table 1 shows participants’ self-perception ratings before and after viewing the videos. Data from the two videos are combined. After viewing the videos, participants rated themselves as looking less anxious than they had predicted, felt that their feared catastrophes occurred to a lesser extent, and rated their overall performance as better than they had anticipated. Table 1 also shows comparable data from McManus et al. (2009). Inspection of effect sizes shows that the beneficial effects of video feedback in the present study were between 38% and 75% larger, depending on the measure used.

**Table 1:** Comparison of Participants’ Ratings of What They Predicted They Would See With What They Actually Saw on the Video for the Present Study and McManus et al. (2009)

*Measure*	*N*	*Predicted Mean* (*SD*)	*After Viewing Mean* (*SD*)	*Effect sizeCohen*’*s d*	*t*
*Present study*					
Look anxious (0–100)	45	55.14 (16.73)	22.89 (18.35)	1.65	11.07***
Mean social fear belief (0–100)	47	47.99 (14.12)	14.60 (14.60)	2.35	16.12***
Overall performance (0–100)	29	53.16 (13.75)	75.91 (14.65)	− 1.78	− 9.60***
Composite score (0–100)	47	50.95 (13.56)	20.47 (15.02)	2.15	14.73***

*McManus et al.* (*2009*)					
Look anxious (0–100)	17	51.18 (18.90)	29.32 (13.52)	1.14	4.69***
Mean social fear belief (0–100)	17	45.09 (18.04)	18.75 (14.28)	1.70	7.0***
Overall performance (0–100)	17	48.13 (17.07)	63.13 (14.51)	− 1.02	− 4.08***
Composite score (0–100)	17	49.46 (15.58)	28.15 (11.70)	1.55	6.37***

*Note*. n = number of participants with paired data. Only a subset of participants were asked to rate their overall performance by their therapists. The composite score was based on the mean of all paired variables that were available for each participant. n = 17 for McManus et al. (2009) as this is the number of individuals who were tested in the same sequence as in the present study, e.g. self-focused attention condition followed by focusing externally. *p < .05, **p < .01, ***p < .001.

To estimate the consistency with which video feedback improved patients’ self perception, a composite score was calculated in line with McManus et al. (2009). The composite score was the mean of the ratings of anxious appearance, social fears and overall performance. Internal consistency of the composite score was acceptable (α = 0.75). Ratings of overall performance were reversed so that higher ratings indicated a more negative appearance/performance on all variables. For 98% of patients their ratings after video feedback were less negative and more favorable than before viewing the video. Table 1 shows the composite scores.

#### Impact on Social Anxiety

In order to assess the impact of video feedback on participants’ subsequent social anxiety, we compared participants’ scores on the LSAS at the start of the video feedback session with their scores 1 week later. To determine whether any change was more than one might expect from the passage of time alone, we compared change that occurred during this week with change that occurred in two other time periods. The first was the interval between the initial assessment interview and Session 1 (usually 2 weeks). This interval provides an estimate of what happens with no intervention. The second interval was that between Session 1 and Session 2. In Session 1 therapists develop a personal version of the Clark and Wells (1995) model and socialize patients to therapy without trying to change beliefs and behaviors. This interval therefore controls for the effects of therapist attention per se.

Table 2 shows LSAS scores at the beginning of the assessment interview; Session 1 (developing an individual formulation of the SAD); Session 2 (self-focus and safety behaviors experiment); Session 3 (video feedback); and Session 4 (1 week after video feedback). A repeated measures ANOVA was used to analyze the data. Mauchly’s test indicated that the assumption of sphericity had been violated, χ^2^(5) = 28.45, *p* < .001, therefore degrees of freedom were adjusted using the Greenhouse–Geisser correction. The results show that the LSAS score differed between the sessions, *F*(2.84, 124.82) = 15.37, *p* < .001. Pairwise comparisons between sessions 3 and 4 showed that there was a significant reduction in LSAS scores (from 73.4 to 65.5) in the week following the video feedback session (*p* = .003). By contrast, there were no significant changes in LSAS in the weeks following either the initial assessment (*p* = 1.00) or developing the individualized cognitive model (Session 1; *p* = .7).

**Table 2:** Means and Standard Deviations of Scores on the Liebowitz Social Anxiety Scale at Baseline Assessment; Session 1 (Drawing out the Model); Session 2 (With Self-Focus and Safety Behaviors Experiment); Session 3 (Video Feedback); and Session 4 (1 Week After Video Feedback)

Measure	Baseline assessment M (SD) n = 47	Session 1 M (SD) N = 45	Session 2 M (SD) N = 47	Session 3 M (SD) N = 47	Session 4 M (SD) N = 47	Time main effect F (3, 125)
LSAS	79.26 (17.56)	79.13 (18.56)	76.15 (20.18)	73.47 (20.85)	65.45 (22.74)	15.37, *p* < *.001*

### Discussion

Our present findings confirm and extend those reported by McManus et al. (2009). Video feedback in Session 3 of a course of CT-SAD was a highly effective intervention for changing negative self-perceptions and was associated with a significant reduction in social anxiety in the following week. The reduction in social anxiety was greater than in two similar time intervals earlier in the course of therapy, suggesting that video feedback has a specific effect, over and above therapist attention, although this result should ideally be confirmed in a between-subjects randomized allocation design.

McManus et al. (2009) found that video feedback had remarkably consistent effects with 94% of participants showing an improvement in their self-perception. This consistency was replicated with 98% of patients in the present study stating that they came across to others more favorably than they had predicted, once they had viewed the video. Inspection of effect sizes indicates that the magnitude of the changes observed with video feedback in the present study were even greater than in McManus et al. (2009). As patient selection criteria were similar in the two studies, this suggests that our refinements in setting up, viewing, and discussing video feedback may have further enhanced the potency of the technique. In the next section we summarize the procedures we have so far found helpful for setting up and implementing video feedback, both within the standard video feedback session that occurs early in cognitive therapy and for its more general use throughout the course of treatment.

## Clinical Guidelines for Conducting Video Feedback in CT-SAD

Our results suggest that video feedback is an excellent technique for helping patients to correct negative self-images and to also gain insight into the way in which their safety behaviors appear to others. There are many opportunities to use it during a course of cognitive therapy, both in the therapy office and also outside of the office when conducting behavioral experiments in the real world. Typically, the first time it is used is in Session 3 when therapist and patient have the opportunity to observe the two social interactions that form part of the self-focused attention and safety behaviors experiment in Session 2. The initial messages that patients learn from this experiment are that self-focused attention and safety behaviors make the problem worse, not better. In particular, they tend to increase anxiety, make it more difficult to focus on the interaction, and give patients enhanced access to internal information (negative images and feelings) that lead them to think that they are coming across to other people more poorly than they really are. The additional messages that patients often get from viewing the videos include: (a) that they come across more favorably than they think in both conditions; (b) some of the aspects of their behavior that they do not like are the unintended, observable consequences of their safety behaviors rather than an intrinsic feature of themselves. When used at other times in therapy, video feedback has a similar function. It also is a very good way of helping patients discover that they are less the subject of other people’s critical attention than they think.

When video feedback is used, it is necessary to pay attention to the way in which the video recording is set up, how the patient is prepared in advance of viewing the recording, and how the video is subsequently viewed and discussed. With suitable attention to each of these aspects, it is often possible to overcome the substantial processing biases that have prevented patients from overcoming their negative self-perceptions before they entered therapy.

### Setting up the Recording

#### Using the Video Camera

As patients with social anxiety can become quite self-conscious about being recorded, it is best to make video recording a routine aspect of therapy, rather than something that is just introduced on an occasional basis for video feedback. We routinely record all therapy sessions using a small domestic video camera that is unobtrusively placed on a bookshelf at right angles to the therapist and patient’s eye line, so it is not in the normal field of view. In the initial assessment interview we explain to patients that we find it helpful to view sessions afterwards in order to reflect on progress and plan future interventions. We request written permission to make the recordings for this purpose. We also encourage patients to take audio recordings of the sessions for them to review afterwards, as we find this is a very good way of maximizing learning. Once permission for the recordings has been obtained, the videos can subsequently also be used for video feedback when therapist and patient together think this might be useful.

As well as making the video less intrusive, routinely recording all sessions allows one to capitalize on unplanned therapy events that can be immensely informative. For example, when talking about a topic in the session patients may spontaneously mention that they feel they blushed a lot, had a panic, stuttered, or talked nonsense. In each case they can be asked to specify how they think they looked or sounded, before comparing their prediction with what was captured on the video. A similar process can be applied to the therapist’s behavior. For example, the patient may feel that one must always be perfectly fluent in one’s speech in order to be accepted. Being aware of this belief, the therapist may pause for a while in mid-sentence before carrying on or may start one sentence and then move onto another without completing the first sentence. Chances are that the patient is unlikely to have noticed this and will be surprised to discover that it happened. Reviewing the video with the therapist afterwards helps the patient see that the dysfluency had no real significance, even though the patient would have felt it was a serious social mistake if she had done it herself.

One of the main aims of video feedback is to allow patients to see their behavior in context. For this reason, when videotaping an interaction it is best to show both people in the interaction, rather than zooming in on the patient. The latter tends to make it difficult for the patient to avoid feeling self-conscious when subsequently watching the video, and also prevents an appreciation of the true significance of behaviors. For example, patients who are concerned about fidgeting might notice their hands or feet moving in a zoomed-in shot and think this indicates that they are very fidgety. However, in a zoomed-out shot they are likely to see that the other person is also moving to a similar extent.

It is important to elicit patients’ main concerns before setting up the video as knowledge about these concerns may have implications for the way in which the video is set up. For example, with patients who are concerned about blushing, it is important to have a color chart or other objects in the field of view that show different shades of red. This is not usually explained to the patient in advance (as it would make them excessively self-conscious). However, if they feel that they do blush during the recording they can subsequently be asked to point to the shade of red that they think matched the blush. Invariably they point to a much darker shade, which is a wonderfully graphic way of helping them discover that their blush is less severe than they feel.

#### Other Participants Taking Part in Social Tasks

During a course of CT-SAD patients are likely to have multiple interactions with other people in therapy sessions (conversations with a stranger, a presentation to a small audience etc.). As well as viewing the video of such interactions with their therapist, patients can also benefit from written feedback from the other participants (“confederates”) in the interaction. In order for this feedback to meaningfully reflect everyday life, it is important that the confederates are not informed about the patient’s personal fears (e.g., “I’ll sound stupid”; “I’ll have nothing to say”; “My lip will tremble”) as this would not be information that other people would have in a routine conversation. The confederate is encouraged to treat the patient like anyone else they would meet in life outside of the therapy session rather than someone they are trying to scrutinize.

Behavioral experiments conducted outside of the office but in a public space can easily be recorded on a smartphone, tablet, or other domestic camera so that video feedback can be used to enhance the value of these exercises. The principles for setting up and viewing such out-of-the-office videos are essentially the same as those that apply to in-office videos.

### Preparing to View the Video

#### Identifying Patients’ Predictions in Advance of Viewing

Before viewing a video, it is important to identify patients’ predictions of what they think they will see and to get them to visualize what these things look like. This provides them with the maximum opportunity to see the difference between their self-perceptions and reality.

Patients are asked to rate (on 0–100 scales) the extent to which they thought their feared catastrophes occurred (“*How anxious do you think you looked*?” “*How boring*?” “*To what extent to you think you sweated*?” etc.) and to indicate how they think that will look. They should be as specific as possible. For example, if somebody says “*I will look in a state*—*just awful*,” we would want to elicit a more specific description of how they think they will look on video so that this can be compared to the actual video image. For situations like shaking, blushing, underarm sweating, and dysfluent speech, it is helpful to ask the person to demonstrate on video how they think it looked—for example, by intentionally shaking one’s hand or lips, pointing to the relevant shade of red in a color chart, indicating the size of a sweat patch, or re-creating a pause—so that this can be compared with how it actually looked in the original video recording.

Once patients’ predictions have been clearly articulated, it is often helpful to ask them to close their eyes and create their own internal video by visualizing how they think they will appear. It is sometimes also useful to ask people to write short notes on how they think they will appear.

#### Preparing an Unbiased Mode of Viewing

It is common for patients to reexperience some of their anxious feelings while watching the video. These feelings may influence their perception of the video. For example, if they feel shaky they may see shaking in the video that would not be seen by others. To overcome this problem, we explain to patients that how one looks and how one feels may not be the same but it is impossible to discover this unless the two are kept separate. In order to do this, patients are asked to view themselves in the video as though they are watching a stranger, only making inferences about how they appear by using what they see and hear on the video, ignoring their feelings. To help them do this, we may encourage them to imagine they are watching a television show. When discussing the video with the therapist, we may ask them to refer to themselves as “that person” or to give themselves a different name.

Some patients reexperience feelings from socially traumatic memories (such as being laughed at, ridiculed, or bullied) while watching the video. These feelings may also distort their perception of the video. If the therapist and patient have identified this problem, patients can be asked to specifically look for things in the video that are inconsistent with the past social trauma to help them clearly distinguish between then and now (e.g., focusing on everything about the people they are currently interacting with, which is different from the people involved with the traumatic experience).

Some people find that it is very difficult not to turn on their habitual self-critical commentary when watching themselves. After discussing how this can take them away from what actually happens on the video, it can be useful to ask them to watch the video from a more compassionate stance, perhaps as they would if they were watching a close friend or somebody they like and respect. They can be asked to recall the last time they had a conversation with this friend and to consider how they would view their friend:

THERAPIST: How do you listen when your friend Alex is talking? Do you just go with the flow of what he says or do you zoom in on how he says every word and ask yourself, “*How boring does Alex sound*?” “*How weird does he look*?” [use patient’s own beliefs]

PATIENT: (laughs) No! I just go with the flow.

THERAPIST: Ok, we would like you to watch the people on the video in a similar way.

Some people find that when they hear the sound of their own voice this automatically triggers the similar sounding self-critical commentary that they normally have in social situations. If the person is highly self-critical, to prevent this mode being activated as soon as the video starts, it can be helpful to watch the first 30 seconds or so with the sound off. This will help them to see that they look as normal as anybody else on the video. When the sound is then turned on, they are in a more appropriate cognitive set. A similar maneuver can be used for people who are highly critical of their physical appearance and find it difficult to look at anything else in the video. For these people, the therapist may initially cover up the patient’s image and let them focus on how other people are responding to them. After a minute or so, they can also be revealed.

There is a risk that people may selectively zoom in on themselves looking for any imperfection, rather than watching the interaction in context. To avoid this, the therapist may say something like: “*Imagine you walk into a coffee shop and see a conversation happening*, *look at the whole group*, *not just one person.*”

### Viewing and Discussing the Video

Once the patient has clearly articulated their negative self-image and they have been carefully prepared for viewing the video, therapist and patient watch it together. Sometimes the whole interaction is initially viewed without pausing. For highly self-critical patients it can be helpful to pause early in the viewing to check the following: “*Are you watching that person (therapist points to the patient in the video) as you would anyone else*, *or are you watching as your worst critic*? *Are you looking at other people and how they respond*, *or just focusing on* *that person*?”

#### Rewinding the Video to Capture Key Moments

Once the video has been watched all the way through, it can be very helpful to rewind the video to look at particular moments that have significance to the patient. For example, rewinding to the moment when the person thought they had a panic attack, when they thought they paused for a long time, looked particularly anxious, looked fidgety or felt that they sweated. They can then be asked to compare how they looked at that moment with their expectation. Other helpful questions may include: “*Does the other person seem to have noticed*?” “*Are they reacting as if they have seen a big mistake*?” “*Do the people onscreen look markedly different from each other*?” “*Is the other person also moving their legs and fidgeting*?” “*If an alien was looking at this would they think one of these people looked really odd*?” As the therapist will be aware of the patient’s own self-images and has also seen how they actually behaved, the exact choice of questions will be determined by the therapist’s goal of helping the patient see which aspects of their self-image are distorted.

#### Gaining Insight Into the Impressions Safety Behaviors Convey to Other People

Patients are often unaware of the way that their safety behaviors appear to other people. Video feedback provides an ideal opportunity for them to gain insight, which in turn can help motivate them to drop the safety behaviors. For example, a patient who was concerned that his colleagues might see his hand shaking while drinking a beer often turned his back to his colleagues before taking a sip. This made him feel less self-conscious and so seemed a good way of coping with the situation until he saw what it looked like on the video. He then realized it will have appeared odd and may have conveyed to his friends that he was not really interested in them, when the exact opposite was the truth. As turning his back was an intentional strategy, he was able to choose not to do it in the future. Similarly, a patient who was concerned that other people might think she was stupid tended to run through a preprepared list of topics during conversations and to be distracted from the conversation by mentally monitoring how she thought she was coming across. On viewing the video, she realized that she was conveying the impression that she was not interested in other people and was just giving them a lecture. This was the opposite of the impression that she wanted to convey, so she experimented with just saying what came into her head and responding spontaneously to what people said. When she watched this on the video she realized that dropping her safety behaviors allowed her to come across to others in the open and friendly manner that she wished.

#### Comparing Ratings Before and After Viewing the Video

A key aspect of video feedback involves comparing patients’ ratings of how they thought they would appear with how they actually appeared once the video has been viewed. This comparison usually involves looking at all the specific predictions that the patient made. The initial 0–100 ratings that patients made in advance of viewing the video are compared with their ratings of the same concerns (looking anxious, sounding boring, etc.) after they have watched and discussed the video. To facilitate comparison a two-column table is constructed. Once the discrepancies have been tabulated, the patient is asked:

THERAPIST: What do you notice when we compare these two sets of ratings?

PATIENT: I look so much better than I thought I was going to. I look OK, not really that anxious, even though I felt it.

THERAPIST: What does that tell you about how visible your anxiety is?

PATIENT: Maybe it isn’t that visible to others. Maybe I come across OK.

THERAPIST: So if your feelings aren’t that visible, are they a reliable judge of how you come across?

PATIENT: No, I guess not.

THERAPIST: So next time you are in a social interaction and feel you are coming across badly, you may want to bring to mind the image of how you actually looked on the video.

#### Eliciting Feedback From Other People

It can be very helpful to supplement video viewing with feedback to the patient from other people who might have been involved in an interaction. We routinely do this for the self-focused attention and safety behaviors experiment. After they have participated in *both* conditions, confederates are asked to think back to each interaction in turn and to provide feedback. We find a two-sided form, handed to the confederate as they leave the therapy room, helpful. The first side is largely blank. Confederates are asked to write a few brief notes about their general impressions. What tends to happen is that confederates mention specific points that make it clear they were interested in the patient and noticed various things about them. But what they noticed was mainly what was talked about, not the specific fears that the patient had (such as “my lip was shaking”). The second side covers the patient’s specific predictions (such as “I’ll sound boring”) and the confederate is asked to rate on 0–100 scales the extent to which this was true. Usually the confederates’ ratings are similar to the patients’ ratings after they have viewed the video, but sometimes the confederate is even more positive. When this happens it can be useful to discuss with the patient why the confederate may have been more positive: “*Is it possible that your ratings are still partly influenced by your private feelings*? *This may be information that nobody else could have.*”

Therapists should use their discretion in deciding whether it is helpful or necessary to supplement video feedback with feedback from others. Most often we present feedback from others after patients have had a chance to view and discuss their video and it is essentially used as a way of further confirming the conclusions they have already reached. However, if the feedback is very positive and the therapist thinks that the patient’s self-criticism may make it difficult for them to view the video objectively, it can be useful to show the other person feedback first. This helps to establish a different cognitive set for viewing the video.

Our data (see above) indicate that almost all (98%) patients view themselves more positively after viewing the video if the experience is set up and discussed in the manner presented here. In rare instances where that does not happen, it can be useful to focus the patient’s attention on how others in the video are reacting to them. This can help them see that features that remain prominent in their own mind have less significance to others. Feedback from the confederate is also helpful in such instances.

#### Freezing the Moment of Disconfirmation and Consolidating Learning

**Figure 1:**
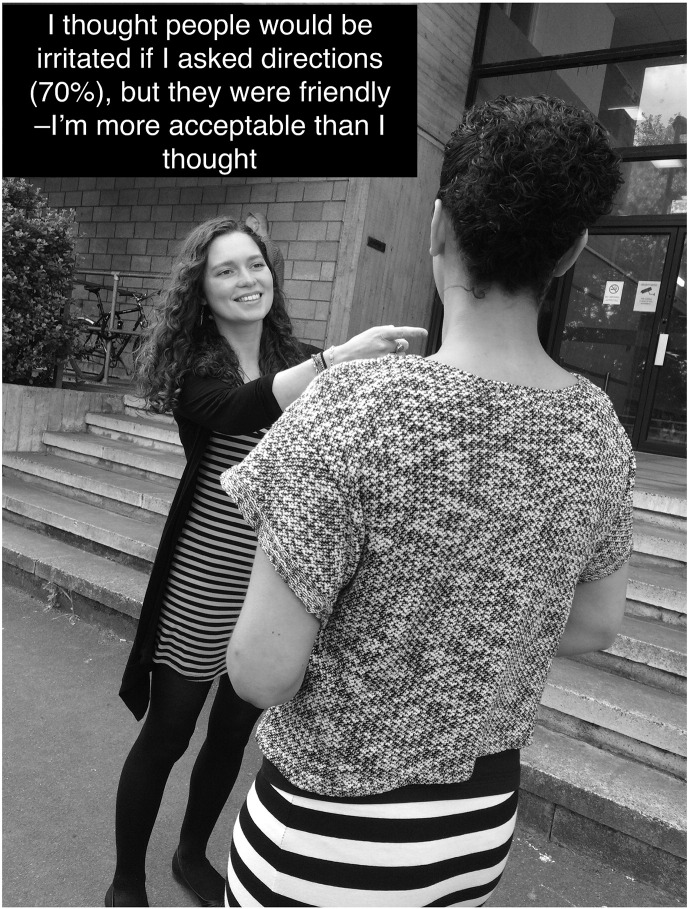
Flashcard demonstrating a stranger’s kind response when approached by the patient who had predicted rejection.

The aim of video feedback is to help patients see that they come across to others much better than they think. There are some moments in a video that illustrate this point more clearly than others. As video is a moving image, these moments can pass in and out of consciousness quite quickly. An excellent way of overcoming this problem is to capture the moment of disconfirmation as a still image. This can be done either by taking a still from the video or by taking a separate photograph. The latter is particularly useful in out-of-the-office behavioral experiments. For example, a patient reported feeling self-conscious when walking in the street even when not interacting with other people. She felt that people were likely to be hostile and predicted that if she asked someone for directions they would respond in an irritated manner, at the very least. She agreed with her therapist that she would test this out by stopping passersby and asking them the directions for the nearby rail station. The therapist accompanied and discretely took photographs on her mobile phone. The image reproduced in Figure 1 captures the moment when her negative prediction was convincingly disconfirmed: the stranger smiles and is helpful when she asks for directions. She pinned the image to her bulletin board at home to remind herself how people really respond to her.

Still images involving other people, like the photograph in Figure 1, can be a helpful way to demonstrate to the patient that their feared concerns (I was boring, I looked sweaty, I looked shaky, I had nothing to say) were not as noticeable to others as they thought, or that even if others did notice, they did not react as negatively as the patient feared. Capturing these moments of disconfirmation can be particularly powerful when the patient feels their feared concern happened naturally (e.g., they forgot what they were saying, they naturally blushed mid-sentence). It can also be helpful for decatastrophizing experiments, where patients purposefully perform a feared concern in order to discover whether others react in the disapproving way they expect (such as adding water to their underarms to create the appearance of sweating, or purposefully trembling their hand when talking to a stranger). This can help patients to realize that they are much less the subject of other people’s critical attention than they initially thought.

**Figure 2:**
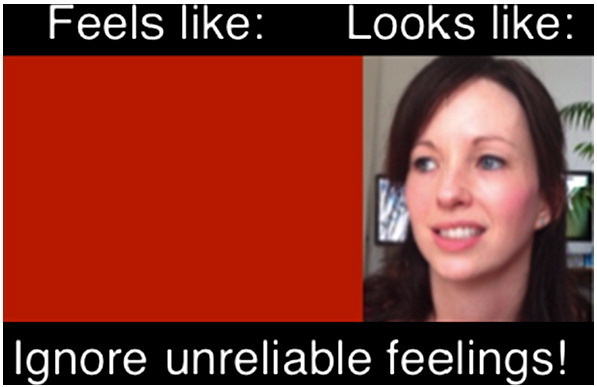
Example of a flashcard capturing the moment of disconfirmation for a patient who worried about blushing.

Still images can also be a wonderful way to capture moments when patients realize they come across much better than their internal feelings and self-perceptions tell them they do (e.g., I look bright red, panicky, have wide scared looking eyes). For example, a patient reported feeling she blushed 80% red while giving a presentation to a small audience during her therapy. Prior to viewing the video, she was asked to select the shade of red she felt she blushed at this moment using a color chart. When a still image was captured from the video at the moment she felt she blushed 80%, the color she selected from the color chart was held next to this image, providing a clear disconfirmation of her belief. A second image was then created: this contained the still taken from the video at the worst moment for the patient (when she felt she blushed red) side-by-side with the block of color she predicted she blushed from the color chart. The image reproduced in Figure 2 illustrates the contrast between the patient’s feelings and the reality. She kept this photo on her mobile phone and looked at it over the week whenever she felt she was blushing, as a reminder that “*My feelings are not as visible as I think.*”

The contrast between two images that depict how patients feel and how they actually looked on video (as demonstrated in Figure 2) may seem stark to an objective observer. However, some patients who reexperience strong anxious feelings and/or find it hard to switch off their habitual self-criticism when viewing images may find it difficult to perceive contrasts that would be apparent to other people who did not experience their feelings or levels of self-criticism. In these instances, we have sometimes found it helpful to edit the image by removing identifiable features of the patient and isolating only the part of the person that they were most concerned about (e.g., showing a portion of their cheek that they felt went bright red; their smile that they felt looked like a grimace; their underarm that they believed was dripping with sweat, etc.). This isolated feature in still image captured from the video can then be compared to a visual calibration obtained before viewing. Removing identifiable features of the patient can help prevent the projection of their feelings and self-criticism into the image, and help them perceive the contrast between their self-perception and reality.

**Figure 3:**
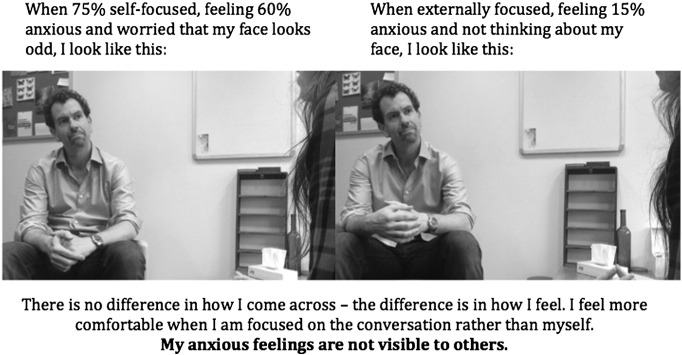
Flashcard to show two different moments during a session illustrating that a patient’s more anxious moment was not noticeable to others.

Capturing two still frames from the video at different time points can also be another way to illustrate that patients’ internal feelings were not as noticeable as they thought. For example, one patient became extremely self-focused during a conversation with a confederate in therapy. He felt highly anxious at that moment and worried that his face looked odd. He and his therapist were able to isolate the moment on video and to also capture a still image from another part of the conversation when he did not feel particularly anxious, and was predominantly externally focused (see Figure 3). The patient was amazed to discover that he couldn’t see any difference between the two images. This helped him realize that his feelings are largely private.

#### Creating a Still Image Flashcard

In order to abstract key principles fitting the cognitive conceptualization of the patient’s social anxiety, and to consolidate and generalize learning, the patient and therapist may add some informative text to still images captured from the video (e.g., “*I felt 80*% *anxious but I don*’*t look it*—*my feelings aren*’*t visible*”; “*I worried other people would laugh at me but they were friendly*, *this shows I*’*m acceptable*”). Adding some of the key learning points in the patient’s own words, either handwritten on a printed copy or typed onto an electronic image that can be saved on a smartphone or tablet, can act as a powerful flashcard that the patient can use as a reminder the next time they enter a stressful situation.

#### Rehearsing the Way They Looked on Video

Negative self-images are often habitual and well rehearsed. For some people it can be helpful for them to intentionally bring to mind the pictures of how they really appeared on video when they appear anxious so these can counteract their habitual negative self-images. They can also remind themselves of how they look before going into a stressful situation.

#### A Demonstration Video Clip

Using the link below, readers can access a 7-minute video clip (Video 1) that illustrates *some* of the procedures described above. The role-played clip is based on a real cognitive therapy session that took 90 minutes. The clip illustrates: (1) Identifying a patient’s predictions in advance of viewing a video; (2) Preparing the patient to view the video; (3) Discussing the video and rewinding to capture key moments; (4) Comparing ratings before and after viewing the video; and (5) Freezing the moment of disconfirmation and consolidating learning.

### Summary of the Clinical Guidelines for Video Feedback

Video feedback is a helpful technique that can be used throughout a course of cognitive therapy to help patients correct their negative self-perceptions and gain insight into the effects of their safety behaviors. Unfortunately, social-anxiety-related processing biases can make it difficult for people with SAD to see the difference between their negative self-image and what is actually shown on the screen. To reliably overcome these biases, particular attention needs to be paid to setting up the video recording; preparing patients to view the video; and subsequently watching and discussing the footage. This clinical guideline has provided details of many of the procedures we have found helpful to maximize the beneficial effects of video feedback. In selecting the most appropriate technique, therapists need to draw on their knowledge of the discrepancy between the patients’ negative self-images and how the patients actually come across. This in turn requires an ability to explore patients’ self-perceptions sensitively and in detail.
